# Antioxidant, Anti-Melanogenic, and Anti-Aging Activities of the Aqueous–Ethanolic Dry Extract of *Rosa lucieae* with Phytochemical Profiling

**DOI:** 10.3390/antiox14101177

**Published:** 2025-09-26

**Authors:** Yun Gyeong Park, Ji-Yul Kim, Seok-Chun Ko, Kyung Woo Kim, Dongwoo Yang, Du-Min Jo, Hyo-Geun Lee, Jeong Min Lee, Mi-Jin Yim, Chul Hwan Kim, Dae-Sung Lee, Hyun-Soo Kim, Gun-Woo Oh

**Affiliations:** 1 National Marine Biodiversity Institute of Korea, Seochun 33662, Republic of Koreakimkw79@mabik.re.kr (K.W.K.);; 2 Department of Seafood Science and Technology, Institute of Marine Industry, Gyeongsang National University, Tongyeong 53064, Republic of Korea

**Keywords:** *Rosa lucieae*, antioxidant activity, tyrosinase inhibition, anti-aging, cosmeceutical ingredient

## Abstract

In this study, the cosmeceutical potential of a 70% ethanol extract of *Rosa lucieae* was investigated as a multifunctional bioactive ingredient. The extract was systematically evaluated for its antioxidant, anti-melanogenic, and anti-aging properties, and was comprehensively phytochemically profiled using ultra-high-performance liquid chromatography–quadrupole time-of-flight mass spectrometry. The analysis tentatively identified 21 metabolites, including phenolic acids (gallic acid, ellagic acid, and corilagin), flavonoids (catechin, rutin, quercetin, hyperoside, and quercitrin), and glycosidic derivatives (e.g., phlorizin), several of which are well-documented for their skin-protective effects. Quantitative measurements confirmed high polyphenol and flavonoid contents, correlating with strong radical-scavenging and reducing capacities in α-diphenyl-β-picrylhydrazyl, 2,2′-azino-bis(3-ethylbenzothiazoline-6-sulfonic acid, as well as ferric ion reducing antioxidant power assays. Moreover, the extract inhibited tyrosinase activity and 3,4-dihydroxyphenylalanine oxidation, thereby suppressing melanin biosynthesis. In addition, marked inhibitory effects against collagenase, elastase, and hyaluronidase were observed; these enzymes are critically involved in extracellular matrix degradation and skin aging. Taken together, these results indicate that the biological activities of *R. lucieae* are supported by a diverse polyphenol- and flavonoid-rich chemical profile, highlighting the potential of this plant as a natural multifunctional ingredient for cosmeceutical, nutraceutical, functional food, and preventive healthcare applications.

## 1. Introduction

The *Rosaceae* family comprises one of the largest and taxonomically diverse groups of flowering plants, including more than 90 genera and nearly 3000 species worldwide [1]. This family covers several genera of major ecological and high economic value (e.g., *Rosa*, *Rubus*, *Malus*, and *Prunus*) that are widely exploited in horticulture, traditional medicine, and food industries [2]. *Rosaceae* are particularly rich in phytochemicals (i.e., phenolic acids, flavonoids, and tannins) well-recognized for their antioxidant, anti-inflammatory, and antimicrobial effects [3,4]. Due to this phytochemical diversity, several *Rosaceae* species are considered important sources of bioactive metabolites with pharmaceutical and cosmeceutical potential. *Rosa lucieae* Franch. & Rochebr. ex Crép. of the family *Rosaceae*, often referred to as memorial rose, represents a vigorous climbing shrub characterized by abundant thorns [5,6]. Native to East Asia, including the southern regions of Korea, southeastern China, and parts of Japan, this species is also cultivated extensively for ornamental purposes and ecological resilience [7]. Its ability to persist in harsh coastal environments suggests an adaptive strategy against abiotic stressors (e.g., increased salinity and intense ultraviolet (UV) exposure) [8]. However, compared with other members of the family, studies on the phytochemistry and bioactivities of *R. lucieae* remain limited, necessitating the systematic exploration of its potential in cosmetic and pharmacological applications.

Growing demand for safe, multifunctional, and natural ingredients in cosmetic science has encouraged increased focus on plant extracts with antioxidative, skin-whitening, and anti-aging effects [9]. Within this scope, polyphenols and flavonoids have drawn attention due to their capacity for scavenging reactive oxygen species (ROS), suppressing melanogenesis, and protecting extracellular matrix (ECM) proteins (e.g., collagen and elastin) from degradation [10]. These metabolites not only counter oxidative stress but also affect skin aging- and pigmentation-linked molecular pathways. Flavonoids (e.g., quercetin and kaempferol) reportedly inhibit tyrosinase, thereby reducing melanin formation [11]. In addition, polyphenolic compounds reportedly suppress matrix metalloproteinases (MMPs), enzymes that degrade structural dermal proteins, thereby contributing to the preservation of skin elasticity and firmness [12]. Beyond dermatological benefits, such antioxidant and enzyme-modulating properties of plant-derived polyphenols and flavonoids also highlight their potential in nutraceutical and preventive healthcare applications.

Oxidative stress is considered a central mechanism underlying both skin aging and pigmentation disorders. ROS (e.g., superoxide anion, hydroxyl radical, and hydrogen peroxide) can initiate molecular damage, activate MMPs, and induce inflammatory responses [13]. External environmental factors, primarily UV irradiation and air pollutants, further intensify ROS generation, leading to collagen and elastin breakdown, skin barrier integrity impairment, and melanogenesis stimulation [14]. These events accelerate visible skin alterations including wrinkles, sagging, and hyperpigmentation. Central to these pathways are several key enzymes (e.g., tyrosinase, collagenase, elastase, and hyaluronidase) responsible for melanin biosynthesis, dermal matrix degradation, and moisture loss [15,16,17]. Therefore, antioxidant-rich botanical extracts are widely investigated for dermatological applications, especially for their ability to neutralize ROS and inhibit aging-related enzymatic activity. Identification of both effective and safe natural inhibitors remains essential not only for developing multifunctional skincare agents but also for advancing applications in dermatology, preventive healthcare, and functional foods.

Although antioxidant and anti-inflammatory activities have been characterized in other *Rosa* species such as *Rosa rugosa* and *Rosa canina*, information on the bioactive profile of *R. lucieae* remains insufficient [18,19,20]. These related species displayed notable skin-protective effects, attributed mainly to their phenolic acid, flavonoid, and essential fatty acid content. *R. lucieae* is naturally distributed along littoral sandbanks and rocky seashores, environments characterized by exposure to salt spray and strong UV radiation, which may drive the production of unique secondary metabolites with strong biological activities. [8,21]. To address this knowledge gap, in this study, the antioxidant, anti-melanogenic, and anti-aging properties of a 70% ethanol extract of *R. lucieae* were evaluated, and comprehensive phytochemical profiling was conducted using ultra-high-performance liquid chromatography–quadrupole time-of-flight mass spectrometry (UPLC-Q-TOF/MS). The aim of this study was to integrate functional assays with metabolite identification in order to not only elucidate the biological potential of this plant but also identify the chemical constituents responsible for its potential activities, thereby reinforcing its application as a multifunctional natural ingredient.

## 2. Materials and Methods

### 2.1. Materials

Gallic acid, rutin, aluminum chloride (AlCl_3_), kojic acid, arbutin, mushroom tyrosinase (EC 1.14.18.1), L-tyrosine, L-3,4-dihydroxyphenylalanine (L-DOPA), epigallocatechin gallate (EGCG), oleanolic acid, 6-O-palmitoyl-L-ascorbic acid, 2,2′-azino-bis(3-ethylbenzothiazoline-6-sulfonic acid) (ABTS), ethanol (EtOH), methanol (MeOH), dimethyl sulfoxide (DMSO), distilled water (DW), Trolox, ascorbic acid, and potassium persulfate were obtained from Sigma-Aldrich (St. Louis, MO, USA). 2,2-Diphenyl-1-picrylhydrazyl (DPPH) was purchased from Wako Pure Chemical Industries (Osaka, Japan), and Folin–Ciocalteu phenol reagent was sourced from Merck Chemicals (Darmstadt, Germany). All other reagents and solvents were of analytical grade. Absorbance and fluorescence were measured using a SpectraMax i3x microplate reader (Molecular Devices, San Jose, CA, USA).

### 2.2. Plant Material and Extraction

Fresh aerial parts of *R. lucieae* were collected in Jeju, Korea, in June 2021 (MABIK NP60220044). The plant material was taxonomically identified and authenticated by Dr. Myung-Ok Moon (Research Institute of Basic Sciences, Jeju National University, Jeju, Republic of Korea), and a voucher specimen was deposited in the herbarium of the National Marine Biodiversity Institute of Korea (Janghang-eup, Republic of Korea). Approximately 100 g of fresh material was harvested, thoroughly washed, and freeze-dried using a lyophilizer (LP200, IlshinBioBase, Dongducheon, Republic of Korea), and the dried material was subsequently pulverized into a fine powder using a mechanical grinder. Extraction was carried out by mixing the 10 g of powdered sample with 70% ethanol at 10:1 (*v*/*v*) and applying ultrasonication at 36 KHz of for 1 h, repeated three times, at room temperature. The resulting mixture was filtered through standard filter paper, and the filtrate was concentrated under reduced pressure at 40 °C and 40 mbar using a rotary evaporator (N-1210 and OSB-2200 water bath, EYELA, Tokyo, Japan) and subsequently freeze-dried for 3 days to obtain a powdered ethanol extract (1.47 g), with a yield of 14.7%, which was used in subsequent experiments. The extract was stored at −80 °C until use in the experiments.

### 2.3. Total Polyphenol and Flavonoid Content

Total polyphenol content of the *R. lucieae* ethanol extract was determined using the Folin–Ciocalteu colorimetric method [22]. In a 96-well microplate, 20 μL of the extract solution was mixed with 100 μL of 1 N Folin–Ciocalteu reagent and the mixture was incubated in the dark at room temperature for 5 min. Subsequently, 80 μL of 7.5% sodium carbonate (Na_2_CO_3_) solution was added and the mixture was incubated in the dark for an additional 20 min. The absorbance at 765 nm was measured using a UV–visible microplate reader and gallic acid (0, 10, 20, 40, 60, 80, and 100 μg/mL) as the standard to generate the calibration curve.

Flavonoid content was measured using the AlCl_3_ reagent [23]. In a 96-well microplate, 100 μL of the extract solution was mixed with 100 μL of 2% AlCl_3_ solution and the mixture was incubated in the dark at room temperature for 10 min. The absorbance at 415 nm was read using a UV–visible microplate reader and rutin (0, 10, 20, 40, 60, 80, and 100 μg/mL) as the standard for the calibration curve.

### 2.4. Liquid Chromatography with Tandem Mass Spectrometry (LC-MS/MS) Analysis of the 70% EtOH Extract from R. lucieae

**Figure 1:**
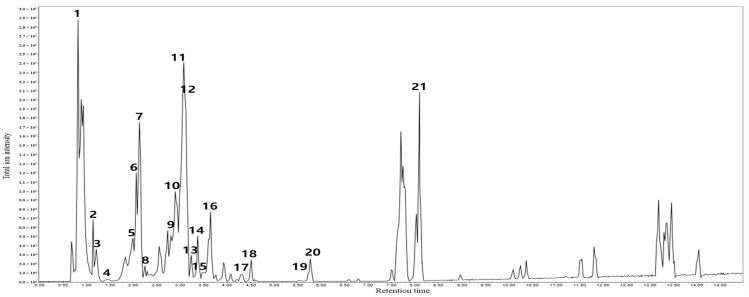
Total ion chromatogram of the 70% ethanol extract of *R. lucieae* obtained by UPLC-Q-TOF/MS in the negative ion mode.

LC-MS/MS was performed using a SCIEX X500R Q-TOF mass spectrometer (Danaher Corp., Washington, DC, USA) in negative ion mode (Figure 1 and Table 1). The UPLC system (ExionLC; Shimadzu, Kyoto, Japan) was equipped with an ACQUITY BEH C18 column (1.7 μm, 2.1 mm × 150 mm; Waters Corp., Milford, MA, USA) at a flow rate of 0.3 mL/min, and injection volume of 10 μL. The mobile phase consisted of acetonitrile (A line) and 0.1% formic acid in water (B line). The organic solvent was increased as follows: 0 min, 10% A; 0–1 min, 20% A; 1–31 min, 98% A; 31–34 min, 98% A; 34–37 min, 10% A; 37–40 min, 10% A. Data analysis was carried out using the MZmine software version 4.5.37 (mzio GmbH, Bermen, Germany). Information-dependent acquisition (IDA) was conducted under negative ion conditions with source and gas parameters as follows: ion source gas 1: 50 psi, ion source gas 2: 50 psi, curtain gas: 30 psi, temperature: 500 °C, CAD gas: 7). The declustering potential, collision energy, CE spread, spray voltage and scan range were set to 80 V, -30 V, −15 V, at 5500 V, and 100–1000 Da, respectively. The non-target search was performed using SCIEX OS (Danaher) installed with an NIST 17 library and compared with a GNPS library.

### 2.5. Antioxidant Activities

DPPH radical scavenging activity was analyzed as follows [24]. Briefly, a series of *R. lucieae* ethanol extract dilutions in DW was prepared. In a 96-well microplate, 100 μL of each sample (0, 1.56, 3.125, 6.25, and 12.5 μg/mL) or ascorbic acid (7 μg/mL) or Trolox (10 μg/mL) at various concentrations was mixed with 100 μL of 0.3 mM DPPH solution. The reaction mixtures were incubated at room temperature for 30 min in the dark, and absorbance at 517 nm was measured using a UV–Vis microplate reader.

ABTS radical scavenging activity was evaluated as follows [25]. A 7-mM ABTS solution was reacted with 2.45 mM potassium persulfate, and the mixture was incubated in the dark for 16 h to generate ABTS radicals. The resulting ABTS radical solution was then diluted with DW until reaching an absorbance of 0.70 ± 0.02 at 745 nm. In a 96-well microplate, 150 μL of the ABTS radical solution was added to 50 μL of *R. lucieae* ethanol extract (0.39, 0.78, 1.56, and 3.12 μg/mL) or ascorbic acid (5 μg/mL) or Trolox (5 μg/mL). After 20 min of reaction at room temperature, the absorbance at 745 nm was recorded using a microplate reader.

The ferric reducing ability of plasma (FRAP) assay was performed using a commercially available kit (MAK369, Sigma-Aldrich) according to the manufacturer’s instructions. Briefly, 10 μL of *R. lucieae* ethanol extract (1.56, 3.125, 6.25, and 12.5 μg/mL) or ascorbic acid (8.8 μg/mL) or Trolox (12.5 μg/mL) at various concentrations was added to 190 μL of the FRAP working reagent, which contained assay buffer, FeCl_3_ solution, and FRAP probe in a 96-well microplate. The plate was incubated at 37 °C for 60 min, and the absorbance at 594 nm was measured using a microplate reader.

### 2.6. Tyrosinase and DOPA Oxidation Inhibition

The tyrosinase inhibitory activity was assessed as follows [26]. Briefly, in a 96-well microplate, 50 μL of *R. lucieae* extract at various concentrations (187.5, 375, 500, and 750 μg/mL), or kojic acid (14.21 μg/mL) or arbutin (272.25 μg/mL) was supplemented with 30 μL of potassium phosphate buffer (pH 6.5), 100 μL of L-tyrosine solution, and 20 μL of mushroom tyrosinase enzyme solution. The reaction mixtures were incubated at 37 °C for 10 min and the absorbance at 490 nm was subsequently measured using a microplate reader.

To assess DOPA oxidation inhibition [27], 50 μL of *R. lucieae* extract (125, 250, 500, and 1000 μg/mL) or kojic acid (71.06 μg/mL) or arbutin (1089 μg/mL) was added to each well along with 30 μL of potassium phosphate buffer (pH 6.5), 100 μL of L-DOPA solution, and 20 μL of mushroom tyrosinase enzyme. The mixtures were incubated at 37 °C for 10 min and the absorbance at 490 nm was measured using a microplate reader.

### 2.7. Anti-Aging Enzyme Inhibition Assays

The collagenase inhibitory activity was measured using a commercial gelatinase/collagenase assay kit (E12055, Thermo Fisher Scientific, Waltham, MA, USA) according to the manufacturer’s instructions. In a 96-well microplate, 80 μL of *R. lucieae* extract (31.25, 62.5, 125, and 250 μg/mL) or EGCG (57.3 μg/mL) or (1,10)-phenanthroline (36 μg/mL) was supplemented with 20 μL of DQ™ gelatin substrate. After 5 min incubation at room temperature, 100 μL of collagenase enzyme was added to each well, the mixtures were incubated for 60 min at room temperature, and fluorescence was measured at excitation and emission wavelengths of 485 and 530 nm, respectively, using a microplate reader.

The elastase inhibitory activity was evaluated using a commercial elastase assay kit (E12056, Thermo Fisher Scientific) according to the manufacturer’s instructions. A 96-well microplate was loaded with 50 μL of *R. lucieae* extract (62.5, 125, 250, and 500 μg/mL) or EGCG (115 μg/mL) or N-methoxysuccinyl-Ala-Ala-Pro-Val-chloromethyl ketone (5 μg/mL), along with 50 μL of 100 μg/mL DQ^TM^ elastin from bovine neck ligament. The mixtures were pre-incubated for 5 min at room temperature and supplemented with 100 μL of 0.5 U/mL porcine pancreas-derived elastase. After incubation for 1 h at room temperature, fluorescence was recorded at excitation and emission wavelengths of 485 and 530 nm, respectively, using a plate reader.

Hyaluronidase inhibition was assessed using a commercial hyaluronidase inhibitor screening kit (MAK458; Sigma-Aldrich) according to the manufacturer’s instructions. In a 96-well microplate, 20 μL of *R. lucieae* extract (7.5, 10, 12.5, and 10 μg/mL), oleanolic acid (80 μg/mL) or 6-O-palmitoyl-L-ascorbic acid (20 μg/mL) was added, followed by 40 μL of hyaluronidase enzyme solution. The mixture was incubated for 15 min at room temperature. Next, 40 μL of working reagent (35 μL assay buffer and 10 μL substrate) was added, and the mixture was incubated for 20 min at room temperature. Finally, 160 μL of stop reagent was added to each well, the plate was incubated for 10-min, and the absorbance at 600 nm was measured using a microplate reader.

### 2.8. Statistical Analysis

The experimental results are expressed as the mean ± standard deviation (SD) from at least three independent replicates. The statistical analyses were conducted using IBM SPSS Statistics software (Version 22.0, IBM Corp., Armonk, NY, USA). *p*-values below 0.01 were considered statistically significant.

## 3. Results

### 3.1. Determination of Polyphenol and Flavonoid Content and UPLC–QTOF–MS Analysis of Rosa lucieae Extract

Polyphenols and flavonoids reportedly neutralize free radicals by donating hydrogen atoms or electrons, thereby preventing oxidative damage to various cellular components (e.g., DNA, proteins, and lipids) [28]. Before antioxidant efficacy was directly assessed, the antioxidant potential of the *R. lucieae* extract was first examined through the quantification of its total polyphenol and flavonoid content. The extract exhibited total polyphenol and flavonoid contents of 224.19 ± 0.73 and 51.57 ± 0.15 mg/g, respectively, indicating strong antioxidant capacities, which align with previous reports of high phenolic content in *Rosaceae* species [29,30].

The tentative identification of 70% EtOH extract from *R. lucieae* was confirmed using high-resolution-Q-TOF/MS in the negative ion mode. The total ion chromatogram of 70% EtOH extract from *R. lucieae* revealed the presence of diverse chemical components (Figure 1). The putative identification of peaks (1–21) was performed by comparing the calculated molecular weights and matched MS/MS fragments with libraries (NIST17 and GNPS) and previous reports (MS/MS fragment ions). Table 1 summarizes the characteristics of the MS/MS spectral data of the 21 peaks (1–21) obtained in the negative ESI mode.

Peak 1 was identified as quinic acid by ion fragments at *m*/*z* 191, 127, 85, and 59 [31]. Citric acid (peak 2) displayed fragment patterns at *m*/*z* 111, 87, 57 [31]. Phenol compounds (gallic acid and ethyl gallate) at peaks 3 and 13 displayed product ions at *m*/*z* 125, 97, and 81 [32]. Furthermore, peak 6 (corilagin) matched MS/MS fragments at *m*/*z* 463, 300, 275, and 169 [33]. A phenolic compound (peak 10; ellagic acid) coincided with major ion fragments at *m*/*z* 300, 283, 245, and 229 [33], and peak 18 (phlorizin) exhibited the same fragment patterns at *m*/*z* 273, 189, and 167 [34]. The main flavonoids derivatives at peaks 4 (gallocatechin), 5 (procyanidin B2), 7 (catechin), 8 (procyanidin C1), 9 (rutin), 11 (hyperoside), 12 (miquelianin), 14 (6″-O-acetylisoquercitrin), 15 (quercitrin), 16 (scutellarin), 17 (prunin), 19 (quercetin), and 20 (tiliroside) were confirmed as previously reported product ion patterns [32,35,36,37,38,39,40,41]. In summary, these results demonstrate that the extract contains a broad range of organic and phenolic acids, flavonoids, and glycosidic derivatives, providing a strong chemical basis for its observed bioactivities.

### 3.2. Antioxidant Properties of R. lucieae Extracts

**Figure 2:**
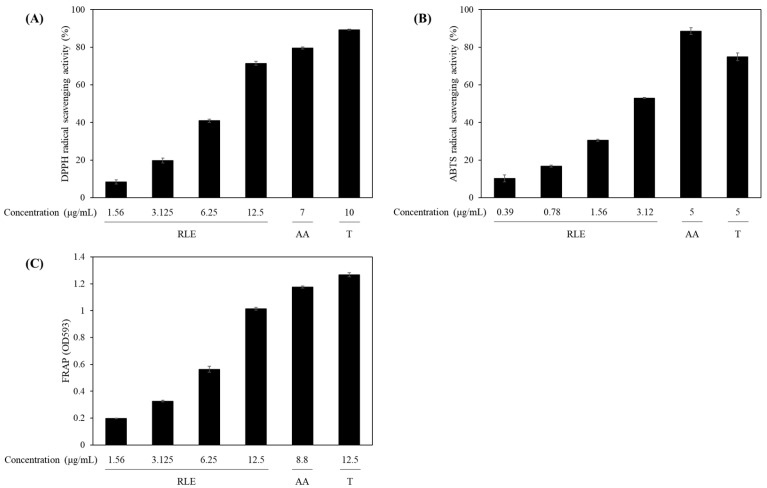
(**A**) 2,2-Diphenyl-1-picrylhydrazyl (DPPH) and (**B**) 2,2′-azino-bis(3-ethylbenzothiazoline-6-sulfonic acid) (ABTS) scavenging activities and (**C**) ferric reducing ability of plasma assay results of the *R. lucieae* ethanol extract. Ascorbic acid (AA) and Trolox (T) were used as reference antioxidants.

Antioxidants in skin care formulations help protect against UV-induced oxidative stress, reduce the signs of aging, and support barrier function [42]. The antioxidant properties of the extract were assessed using DPPH and ABTS radical scavenging assays as well as a FRAP assay. In the DPPH assay (Figure 2A), the extract demonstrated radical scavenging activities of 8.45% ± 1.13%, 19.76% ± 1.29%, 40.91% ± 0.87%, and 71.45% ± 1.03% at concentrations of 1.56, 3.13, 6.25, and 12.5 μg/mL, respectively. The calculated IC_50_ value was 8.47 ± 0.02 μg/mL (Table 2). For the ABTS radical scavenging assay (Figure 2B), the extract displayed scavenging rates of 10.26% ± 1.81%, 16.78% ± 0.49%, 30.56% ± 0.51%, and 53.01% ± 0.33% at varying concentrations of 0.39, 0.78, 1.56, and 3.12 μg/mL, respectively, with an IC_50_ value of 2.91 ± 0.01 μg/mL (Table 2). As positive control, ascorbic acid exhibited scavenging activities of approximately 79.51% ± 0.56% and 88.57% ± 1.77% at 7 and 5 μg/mL, respectively, in the DPPH and ABTS assays, while Trolox displayed corresponding scavenging activities of 89.32% ± 0.26% and 74.92% ± 2.03% at concentrations of 10 and 5 μg/mL, respectively. The *R. lucieae* extract performance in these assay underscores the suitability of this plant as a natural antioxidant ingredient in cosmetic products.

FRAP assay (Figure 2C) was applied to evaluate the electron-donating capacity of the *R. lucieae* extract. This method is based on ferric (Fe^3+^) ion reduction to ferrous (Fe^2+^) ions, providing a direct measure of the overall reducing power of the extract [43]. The FRAP assay demonstrated a concentration-dependent absorbance value increase for the *R. lucieae* extract, with measurements of 0.198 ± 0.002, 0.326 ± 0.006, 0.563 ± 0.023, and 1.013 ± 0.011 at concentrations of 1.56, 3.125, 6.25, and 12.5 μg/mL, respectively. Ascorbic acid and Trolox, used as positive controls, exhibited absorbance values of 1.1764 ± 0.007 and 1.268 ± 0.016 at concentrations of 8.8 and 12.5 μg/mL, respectively. The abundant polyphenols and flavonoids in the *R. lucieae* extract are presumably central to ferric-to-ferrous ion reduction, a well-established antioxidant defense mechanism [44,45].

### 3.3. Anti-Melanogenic Activity of R. lucieae Extracts

**Figure 3:**
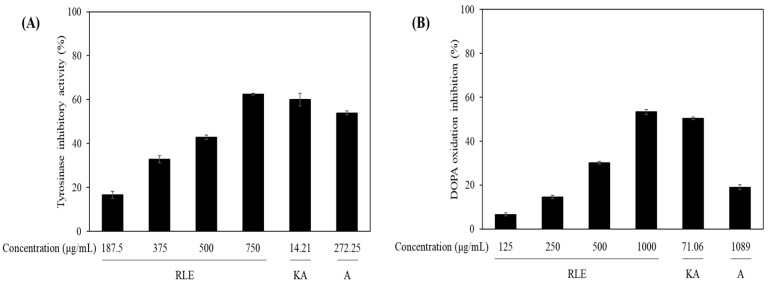
(**A**) Tyrosinase and (**B**) L-3,4-dihydroxyphenylalanine (L-DOPA) oxidation inhibitory activity of the *R. lucieae* ethanol extract. Kojic acid (KA) and arbutin (A) were used as positive controls of tyrosinase inhibition.

Next, the anti-melanogenic activity of the *R. lucieae* extract was evaluated by assessing its inhibitory effects on mushroom tyrosinase activity and DOPA oxidation. Tyrosinase is pivotal for the melanin biosynthesis pathway, and its inhibition is a well-established target for developing skin-whitening agents [46]. The tyrosinase inhibitory (Figure 3A) activity of the *R. lucieae* extract was evaluated at concentrations of 187.5, 375, 500, and 750 μg/mL, yielding inhibition rates of 16.58% ± 1.60%, 32.84% ± 1.67%, 42.91% ± 1.02%, and 62.54% ± 0.44%, respectively, and a calculated IC_50_ value of 591.45 ± 3.46 μg/mL (Table 2). Arbutin and kojic acid inhibition rates were measured as positive controls, yielding 53.97% ± 0.97% at 272.25 μg/mL and 59.99% ± 2.87% at 14.21 μg/mL, respectively. DOPA oxidation inhibition was particularly important due to its essential role in melanin polymerization [47].

In a subsequent DOPA oxidation inhibition assay (Figure 3B), *R. lucieae* extract displayed inhibitory activities of 6.66% ± 0.69%, 14.57% ± 0.82%, 30.23% ± 0.58%, and 53.39% ± 1.11% at concentrations of 125, 250, 500, and 1000 μg/mL, respectively. The IC_50_ value was 918.02 ± 11.42 μg/mL (Table 2). In comparison, arbutin and kojic acid inhibition rates were 19.05% ± 1.12% at 1089 μg/mL and 49.32% ± 0.64% at 71.06 μg/mL, respectively.

### 3.4. Anti-Aging Enzyme Inhibition of the R. lucieae Extract

The anti-aging potential of the *R. lucieae* extract was assessed by evaluating its inhibitory effects on three major skin aging-associated enzymes: collagenase, elastase, and hyaluronidase. These enzymes contribute to the degradation of structural components (including collagen, elastin, and hyaluronic acid) in the dermis [48]. As these three enzymes are central for the breakdown of the dermal matrix and the loss of skin integrity, their inhibition is an important strategy for skin aging prevention.

**Figure 4:**
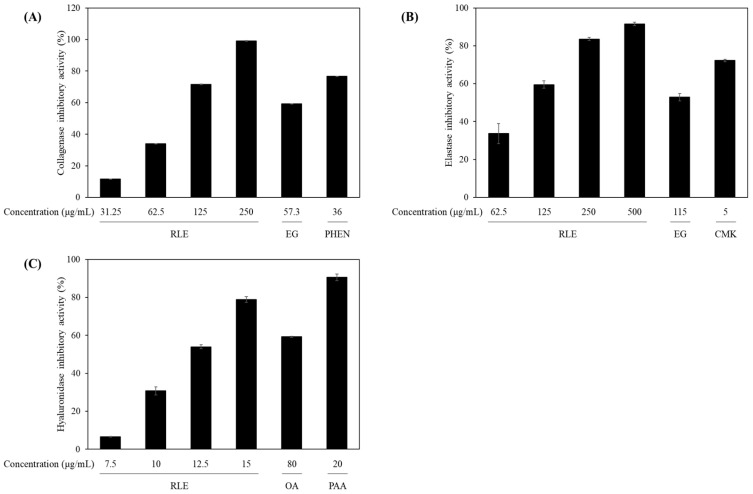
(**A**) Collagenase, (**B**) elastase, and (**C**) hyaluronidase inhibitory activities of the *R. lucieae* ethanol extract. Epigallocatechin gallate (EGCG, EG) and (1,10)-phenanthroline (PHEN) were used as positive control inhibitors for collagenase; EGCG (EG) and N-methoxysuccinyl-Ala-Ala-Pro-Val-chloromethyl ketone (CMK) were used for elastase; and oleanolic acid (OA) and 6-O-palmitoyl-L-ascorbic acid (PAA) were used for hyaluronidase.

In the collagenase inhibition assay (Figure 4A), the ethanol extract of *R. lucieae* exhibited dose-dependent inhibition activity at concentrations of 31.25, 62.5, 125, and 250 μg/mL, with corresponding inhibition rates of 11.61% ± 0.03%, 33.99% ± 0.08%, 71.62% ± 0.16%, and 99.01% ± 0.22%, respectively, and a calculated IC_50_ value of 80.46 ± 0.21 μg/mL (Table 2). As positive controls, (1,10)-phenanthroline and EGCG displayed inhibition rates of 76.63% ± 0.17% and 59.23% ± 0.13% at concentrations of 36 and 57.3 μg/mL, respectively. These results suggest that the extract exerts a strong collagenase inhibitory potential, which might contribute to collagen preservation and the prevention of skin firmness loss and wrinkle formation.

Similarly (Figure 4B), the elastase inhibitory activity of the extract was evaluated at concentrations of 62.5, 125, 250, and 500 μg/mL, yielding inhibition rates of 33.73% ± 5.31%, 59.54% ± 1.90%, 83.55% ± 0.81%, and 91.53% ± 0.84%, respectively, and an IC_50_ value of 96.94 ± 11.81 μg/mL (Table 2). As positive controls, EGCG and N-methoxysuccinyl-Ala-Ala-Pro-Val-chloromethyl ketone exhibited 52.91% ± 1.98% and 72.25% ± 0.7% inhibition at 115 and 5 μg/mL, respectively. These results suggest that the extract effectively inhibits elastase activity, contributing to skin elasticity maintenance and the prevention of sagging.

Furthermore (Figure 4C), the extract displayed marked hyaluronidase inhibitory activity at concentrations of 6.57, 30.72, 53.99, and 78.88 μg/mL, resulting in inhibition rates of 6.57% ± 0.12%, 30.72% ± 2.14%, 53.99% ± 0.98%, and 78.88% ± 1.42%, respectively, and an IC_50_ value of 12.03 ± 0.17 μg/mL (Table 2). As reference compounds, oleanolic acid and 6-O-palmitoyl-L-ascorbic acid showed inhibition rates of 59.31% ± 0.28% and 90.61% ± 1.79% at 80 and 20 μg/mL, respectively. These results suggest that the hyaluronidase inhibitory effect of the extract might help preserve dermal moisture content and improve skin hydration and plumpness.

## 4. Discussion

The observed antioxidant effects could be attributed to the synergistic effect of various bioactive compounds in the extract, including phenolic acids, flavonoids, and possibly other secondary metabolites (e.g., tannins). High polyphenol and flavonoid content in plant extracts reportedly positively correlate with enhanced antioxidant activities, as these compounds effectively scavenge free radicals and chelate metal ions, thereby mitigating oxidative stress [49,50]. In our study, the *R. lucieae* extract displayed high polyphenol and flavonoid content, which paralleled its radical scavenging and reducing activities. Similar associations between phenolic and flavonoid content and antioxidant capacity have been widely reported in the literature. For instance, a study on 12 Indonesian indigenous *Zingiberaceae* herbs revealed that *Curcuma longa*, with the highest total phenolic and flavonoid contents, exhibited the strongest antioxidant activity, underscoring the pivotal role of these phytochemicals in antioxidant efficacy [51]. Similarly, research on *Salvia officinalis* extracts indicated a strong positive correlation between total phenolic and flavonoid contents and antioxidant capacities measured by DPPH, FRAP, and total antioxidant capacity assays, highlighting the significance of these compounds in countering oxidative damage [52]. These results suggest that the rich polyphenol and flavonoid profile of *R. lucieae* extract might substantially contribute to its potent antioxidant properties, thereby reinforcing its potential as a natural ingredient in skincare formulations aimed at mitigating oxidative stress-induced aging of the skin.

Skin whitening remains a major focal point in cosmetic science, especially for individuals aiming to improve overall complexion and achieve a youthful appearance [53]. Although widely used agents (e.g., arbutin and kojic acid) are reportedly efficient in melanin synthesis inhibition, their clinical applications are often limited due to concerns over skin irritation, cytotoxicity, and hyperpigmentation [54,55]. Therefore, demand for safer, natural ingredients that could serve as alternatives to conventional agents is increasing. The bioactive compounds responsible for this activity are likely polyphenols and flavonoids, reportedly chelating the copper ions in the active site of tyrosinase or interfering with the substrate binding of the enzyme [56]. Furthermore, the antioxidant properties of the extract might indirectly contribute to reduced melanogenesis by lowering oxidative stress, which is known to upregulate melanin production via the MITF pathway [57]. This dual mechanism (i.e., direct enzyme inhibition and melanin precursor suppression) supports the inclusion of the extract in cosmetic products aimed at brightening and evening-out the skin tone.

Excessive ROS generation reportedly activates the above-mentioned three key enzymes (i.e., collagenase, elastase, and hyaluronidase) involved in skin aging [10]. The observed inhibitory activities against collagenase, elastase, and hyaluronidase might be attributed to the high polyphenol and flavonoid content in the *R. lucieae* extract. Other polyphenol-rich plant extracts reportedly display similar bioactivities. For instance, *Stenocarpus sinuatus* and *Euphorbia characias* extracts have exerted comparable anti-collagenase, anti-elastase, and anti-hyaluronidase effects, attributed to their high vitamin E and phytochemical levels [58,59]. In particular, the strong hyaluronidase inhibition exerted by the *R. lucieae* extract highlights the potential of this plant to maintain dermal moisture by preventing breakdown of hyaluronic acid, a critical component in skin hydration and plumpness [60]. This property is particularly relevant in cosmetic formulations aimed at alleviating skin dryness and restoring volume. Based on these results, the extract is expected to be a promising multifunctional ingredient for healthcare application, not only due to its antioxidant properties but also through direct collagenase, elastase, and hyaluronidase inhibition.

In parallel with our biological assays, the metabolite profile offers mechanistic insights into the multifunctional activities of the *R. lucieae* extract. Several of the identified compounds are well-recognized for their skin-protective effects. Gallic and ellagic acid are potent ROS scavengers and suppress melanogenesis by inhibiting tyrosinase activity and MITF-related signaling [61,62]. Catechins and procyanidins act as collagenase and elastase inhibitors, thereby limiting extracellular matrix degradation and contributing to dermal resilience [63,64,65]. Flavonols (e.g., rutin and quercetin) exert both antioxidant and anti-inflammatory effects, thereby modulating the MAPK and NF-κB signaling pathways to reduce MMP expression and attenuate inflammatory cascades [66,67,68]. Phlorizin, a dihydrochalcone glycoside, reportedly alleviates UV-induced oxidative stress and improves fibroblast viability, which supports its role in photoprotection [69]. The high radical scavenging and reducing activities observed in the DPPH, ABTS, and FRAP assays correspond to phenolic acid and flavonoid abundance. Similarly, the inhibition of tyrosinase and DOPA oxidation could be explained by the presence of flavonols (e.g., quercetin and rutin), acting as competitive tyrosinase inhibitors. The pronounced inhibitory effects on collagenase, elastase, and hyaluronidase reflect the known anti-MMP and anti-hyaluronidase properties of catechins, ellagic acid, and quercetin derivatives. In summary, the multifunctional activities of *R. lucieae* extract appear to arise not from a single dominant constituent but from the synergistic interplay of multiple phenolic and flavonoid metabolites. The integration of UPLC-Q-TOF/MS profiling with the functional assays presented in this study provides a comprehensive explanation for the biological effects of the extract and reinforces its potential as a valuable natural resource.

## 5. Conclusions

In conclusion, a 70% ethanol extract of *R. lucieae* demonstrated strong antioxidant activity, moderate tyrosinase inhibition, and marked collagenase, elastase, and hyaluronidase suppression, indicating its potential to protect against oxidative stress, pigmentation, and extracellular matrix degradation. UPLC-Q-TOF/MS profiling revealed a diverse phytochemical composition, comprising phenolic acids, flavonoids, and glycosidic derivatives, providing a chemical basis for these multifunctional activities and suggesting the synergistic contribution of multiple metabolites. Taken together, these results highlight *R. lucieae* extract as a promising natural candidate for multifunctional applications in cosmeceuticals, nutraceuticals, and preventive healthcare. Future studies should focus on isolating key active compounds, clarifying molecular mechanisms, and validating efficacy and safety through cellular, in vivo, and clinical investigations.

## Figures and Tables

**Table 1:** Tentative identification of peaks 1–21 using UPLC-Q-TOF/MS analysis.

Peaks	Retention Time	Molecular Formula	Detected Ion [M − H]	Calculated Ion [M − H]	ppm (Error)	Tentative Identification
1	0.83	C_7_H_12_O_6_	191.0548	191.0556	−4.1	Quinic acid
2	1.15	C_6_H_8_O_7_	191.0175	191.0192	−8.8	Citric acid
3	1.22	C_7_H_6_O_5_	169.0132	169.0137	−2.9	Gallic acid
4	1.46	C_15_H_14_O_7_	305.0667	305.0661	1.9	Gallocatechin
5	1.95	C_30_H_26_O_12_	577.1331	577.1346	−2.5	Procyanidin B2
6	2.06	C_27_H_22_O_18_	633.0727	633.0728	−0.1	Corilagin
7	2.13	C_15_H_14_O_6_	289.0701	289.0712	−3.8	Catechin
8	2.39	C_45_H_37_O_18_	865.1962	865.1980	−2.0	Procyanidin C1
9	2.89	C_27_H_30_O_16_	609.1449	609.1456	−1.1	Rutin
10	2.92	C_14_H_6_O_8_	300.9976	300.9984	−2.6	Ellagic acid
11	3.04	C_21_H_20_O_12_	463.0863	463.0877	−3.0	Hyperoside
12	3.10	C_21_H_18_O_13_	477.0656	477.0669	−2.7	Miquelianin
13	3.24	C_9_H_10_O_5_	197.0450	197.0450	0	Ethyl gallate
14	3.40	C_23_H_22_O_13_	505.0978	505.0982	−0.7	6″-O-acetylisoquercitrin
15	3.47	C_21_H_20_O_11_	447.0904	447.0928	−5.3	Quercitrin
16	3.65	C_21_H_18_O_12_	461.0704	461.0720	−3.4	Scutellarin
17	4.03	C_21_H_22_O_10_	433.1129	433.1135	−1.3	Prunin
18	4.53	C_21_H_24_O_10_	435.1291	435.1287	0.9	Phlorizin
19	5.77	C_15_H_10_O_7_	301.0332	301.0348	−5.3	Quercetin
20	5.78	C_30_H_26_O_13_	593.1284	593.1295	−1.8	Tiliroside
21	8.10	C_36_H_58_O_10_	695.3992 [M + FA]^-^	695.4007 [M + FA]^-^	−2.1	Rosamultin

**Table 2:** Half-maximal inhibitory concentration (IC_50_) values of the antioxidant and enzyme-inhibitory activities of the *R. lucieae* ethanol extract.

	Tyrosinase	DOPA Oxidation	Collagenase	Elastase	Hyaluronidase
IC_50_ (μg/mL)	918.02 ± 11.42	591.45 ± 3.46	80.46 ± 0.21	96.94 ± 11.81	12.03 ± 0.17

## Data Availability

The authors confirm that the data supporting the findings of this study are available within the article.

## Abbreviations

The following abbreviations are used in this manuscript:

UV	Ultraviolet
ROS	Reactive Oxygen Species
ECM	Extracellular Matrix
MMP	Metalloproteinases
UPL	Ultra-High-Performance Liquid Chromatography
Q-TOF/MS	Quadrupole Time-of-Flight Mass Spectrometry
AlCl_3_	Aluminum Chloride
L-DOPA	L-3,4-Dihydroxyphenylalanine
EGCG	Epigallocatechin Gallate
ABTS	2,2′-Azino-Bis(3-ethylbenzothiazoline-6-sulfonic acid)
EtOH	Ethanol
MeOH	Methanol
DMSO	Dimethyl Sulfoxide
DW	Distilled Water
DPPH	2,2-Diphenyl-1-picrylhydrazyl
Na_2_CO_3_	Sodium Carbonate
FRAP	Ferric Reducing Ability
IDA	Information Dependent Acquisition
SD	Standard Deviation
Fe^3+^	Ferric
Fe^2+^	Ferrous
